# HDAC3–ERα Selectively Regulates TNF-α-Induced Apoptotic Cell Death in MCF-7 Human Breast Cancer Cells via the p53 Signaling Pathway

**DOI:** 10.3390/cells9051280

**Published:** 2020-05-21

**Authors:** Seung-Ho Park, Hyunhee Kim, Sungmin Kwak, Ji-Hoon Jeong, Jangho Lee, Jin-Taek Hwang, Hyo-Kyoung Choi, Kyung-Chul Choi

**Affiliations:** 1 Department of Biomedical Sciences, Asan Medical Center, AMIST, University of Ulsan College of Medicine, Seoul 05505, Korea; mylove9322@hanmail.net (S.-H.P.); ttlok1816@naver.com (H.K.); bigawa88@naver.com (S.K.); max1431@naver.com (J.-H.J.); 2 Department of Pharmacology, University of Ulsan College of Medicine, Seoul 05505, Korea; 3 Korea Food Research Institute, Wanju-gun 55365, Korea; 07936@kfri.re.kr (J.L.); jthwang@kfri.re.kr (J.-T.H.); 4 Department of Food Biotechnology, Korea University of Science & Technology, Daejeon 34113, Korea

**Keywords:** TNF-α, p53, HDAC3, apoptosis, ERα

## Abstract

Tumor necrosis factor-α (TNF-α) plays a significant role in inflammation and cancer-related apoptosis. We identified a TNF-α-mediated epigenetic mechanism of apoptotic cell death regulation in estrogen receptor-α (ERα)-positive human breast cancer cells. To assess the apoptotic effect of TNF-α, annexin V/ propidium iodide (PI) double staining, cell viability assays, and Western blotting were performed. To elucidate this mechanism, histone deacetylase (HDAC) activity assay and immunoprecipitation (IP) were conducted; the mechanism was subsequently confirmed through chromatin IP (ChIP) assays. Finally, we assessed HDAC3–ERα-mediated apoptotic cell death after TNF-α treatment in ERα-positive human breast cancer (MCF-7) cells via the transcriptional activation of p53 target genes using luciferase assay and quantitative reverse transcription PCR. The TNF-α-induced selective apoptosis in MCF-7 cells was negatively regulated by the HDAC3–ERα complex in a caspase-7-dependent manner. HDAC3 possessed a p53-binding element, thus suppressing the transcriptional activity of its target genes. In contrast, MCF-7 cell treatment with TNF-α led to dissociation of the HDAC3–ERα complex and substitution of the occupancy on the promoter by the p53–p300 complex, thus accelerating p53 target gene expression. In this process, p53 stabilization was accompanied by its acetylation. This study showed that p53-mediated apoptosis in ERα-positive human breast cancer cells was negatively regulated by HDAC3–ERα in a caspase-7-dependent manner. Therefore, these proteins have potential application in therapeutic strategies.

## 1. Introduction

Breast cancer is one of the most predominant malignancies in women. More than 1 million women are diagnosed with the disease worldwide, and it is also the second leading cancer-related cause of death [1,2]. The main signatures of breast cancer cases are sporadic and occur by more complicated factors [3], with the predominant cause of the disease being related to the estrogen receptor (ER) and to estrogen exposure [4,5]. The balance between apoptosis and proliferation in breast cancer cells is very important in determining general growth or repression of the tumors in response to radiotherapy, chemotherapy, or hormonal treatment therapy. In particular, failure of apoptosis is a major predictor of chemotherapy resistance and poor prognosis [6].

The tumor necrosis factor-α (TNF-α), which exists in two bioactive forms—26 kDa and 17 kDa forms, a transmembrane form and a soluble form, respectively—induces cancer cell apoptosis [7,8] by enhancing caspase activity through extrinsic (death receptor signaling) pathways, such as the TNF receptor I (TNFR I), Fas, and TNF-α-related apoptosis-inducing ligand receptor 1 or intrinsic (mitochondrial) pathways mediated by Bad, Bid, and Bax [9,10]. Following activation by TNF-α, Bax translocates to the mitochondrial membrane from the cytosol, where it induces cytochrome c release and caspase activation [11]. Caspases cause the physical and biochemical alterations that occur during apoptosis by catalyzing proteolysis and triggering the cleavage of poly(adenosine diphosphate (ADP)-ribose) polymerase (PARP) by DNA repair enzymes [12,13]. Conversely, TNF-α is implicated in various biological processes, such as inflammation, cell survival, cell proliferation, and cell differentiation, which implies that this molecule is a double-edged sword in cancer treatment [14,15].

Histone deacetylases (HDACs) control gene transcription primarily through histone deacetylation. However, HDACs have several non-histone protein substrates, including signal transducer and activator of transcription (STAT), nuclear factor-kappaB (NF-κB), p21, and p53, which are important transcription factors [16,17,18,19]. Because this altered expression and mutations of HDACs are closely correlated with tumor development and progression, they represent significant therapeutic targets in several human cancers. In particular, the cleavage and inactivation of HDAC3 contribute to the effector caspases’ activation [20]. Furthermore, HDAC3 inactivation leads to the stabilization of p53 through its acetylation, causing its oligomerization and inducing apoptosis [21]. It has also been reported that HDAC3 inhibition decreases the stability of the estrogen receptor-α (ERα) mRNA in ERα-positive human breast cancer (MCF-7) cells [22]. ERα bound to p53 and inhibited its transcriptional functions in ERα-positive human breast cancer cells, as well as in a xenograft mouse model, consequentially impeding p53-dependent apoptosis and cell-cycle arrest [23,24,25]. These findings suggest that cancer cells resist apoptotic cell death, at least in part, through HDAC3-implicated mechanisms, as well as the fact that HDAC3-specific inhibitors in particular will be a new therapeutic strategy for ERα-positive breast cancer. However, the specific mechanism underlying the function of HDAC3 in p53-dependent apoptosis has not been revealed completely.

In the present study, we demonstrated that TNF-α induces the acetylation and stabilization of p53 following caspase-7-dependent HDAC3 cleavage, thus leading to apoptotic cell death in ERα-positive human breast cancer cells (MCF-7 cells). Furthermore, we revealed that ERα is dissociated from the p53-binding element on its target gene, as assessed using a chromatin IP (ChIP) assay, in association with TNF-α-induced HDAC3 cleavage. Finally, we verified that HDAC3-specific inhibition is required for the transcriptional activation of p53 target genes. Our findings highlight the functional significance of HDAC3-dependent ERα repression in TNF-α-induced p53-dependent apoptotic cell death in ERα-positive human breast cancer cells.

## 2. Materials and Methods

### 2.1. Cell Culture and Reagents

MCF-7 and MDA-MB-231 human breast cancer cell lines were purchased from the American Type Culture Collection and were cultured in Dulbecco’s Modified Eagle Medium (DMEM) supplemented with 10% fetal bovine serum (FBS; Thermo-Fischer Scientific, Waltham, MA, USA) and a 1% antibiotic–antimycotic solution (Thermo-Fischer Scientific, Waltham, MA, USA) in a humidified 5% CO_2_ atmosphere at 37 °C. Cells were treated with TNF-α (Sigma-Aldrich, St. Louis, MD, USA) as the indicated conditions. The Lipofectamine 2000 transfection reagent was purchased from Thermo-Fischer Scientific. Z-VAD-FMK, a pan-caspase inhibitor and Z-DQMD-FMK, a caspase-3 inhibitor were obtained from R&D systems (Minneapolis, MN, USA). MS-275, an HDAC1 & 3 inhibitor and trichostatin A (TSA), a pan-HDAC inhibitor were purchased from Selleckchem (Houston, TX, USA).

### 2.2. Cell Viability Assay

Cell viability was determined using the conventional 3-(4,5-dimethylthiazol-2-yl)-2,5-diphenyltetrazolium bromide (MTT) reduction assay. Briefly, 5 × 10^3^ to 1 × 10^4^ cells were seeded per well in 96-well plates, and the cells were further incubated with or without each reagent (as described below and in the Results section) for an additional 24 h. Following treatment of 2 mg/mL of MTT solution for 90 min at 37 °C, absorbance was then measured at 570 nm (Model 550 micro-plate reader; BIO-RAD Laboratories, Hercules, CA, USA). All values were presented as the mean ± SD of three independent experiments.

### 2.3. siRNA Assay

Small interfering RNAs (siRNAs) were obtained from Bioneer Corporation (Daejeon, Korea): HDAC1 siRNA, sense 5′-GAGUCAAAACAGAGGAUGA-3′, antisense 5′-UCAUCCUCUGUUUUGACUC-3′; HDAC3 siRNA, sense 5′-GAGCUUCAAUAUCCCUCUA-3′, antisense 5′-UAGAGGGAUAUUGAAGCUC-3′; p53 siRNA #1, sense 5′-CACUACAACUACAUGUGUA-3′, antisense 5′-UACACAUGUAGUUGUAGUG-3′; and p53 siRNA #2, sense 5′-GAGGUUGGCUCUGACUGUA-3′, antisense 5′-UACAGUCAGAGCCAACCUC-3′. A total of 100 pmol of a non-specific siRNA, the HDAC1 siRNA, or the HDAC3 siRNA, were transported using the Lipofectamine 2000 system into the cells, according to manufacturer’s protocol.

### 2.4. Caspase Activity Assay

TNF-α-induced caspase activities were measured using Caspase-Glo 3/7 and total caspase activity kits (Promega, Madison, WI, USA). Briefly, 4 × 10^3^ cells were plated in a 96-well plate (Lonza, Basel, Switzerland), treated with TNF-α and/or DQMD, and then the 100 μL of assay reagent additionally added. After incubation for 60 min, the luminescence was measured using a plate reader (SpectraMAX 250 Optima; Molecular Devices, Sunnyvale, CA, USA).

### 2.5. HDAC Activity Assays

Histone deacetylase (HDAC) activity assays were performed using a colorimetric system from Biovision (San Francisco, CA, USA) in accordance with the manufacturer’s protocol. To perform assays for specific HDACs—the HDAC1, HDAC2, HDAC3, and HDAC8—each protein was immunoprecipitated in the cell lysates using the antibodies against the respective proteins. Immunoprecipitated complexes were collected with agarose A/G beads (Santa Cruz, Dallas, TX, USA) and after washing with HDAC assay buffer (10% glycerol, 50 mM Tris (pH 8.0), and 0.1 mM ethylenediaminetetraacetic acid (EDTA)) used for assays.

### 2.6. Nuclear Extracts

After this, the trypsinized cells were then carefully transferred and centrifuged at 900× *g* for 3 min at 4 °C, and the cell pellets were used to obtain nuclear faction. Briefly, the cytosolic fraction was removed by Sol A buffer (10 mM KCl, 10 mM Tris (pH 7.4), 0.5% Nonidet *p*-40 (NP-40), and 3 mM MgCl_2_ with a protease inhibitor cocktail) at 1500× *g* for 5 min at 4 °C. The nuclear fraction was obtained from the left pellet with Sol B buffer (0.42 M NaCl, 20 mM Tris (pH 7.9), 10% glycerol, 0.2 mM EDTA, and 2 mM dithiothreitol (DTT) with a protease inhibitor cocktail). The lysates were incubated for 30 min on ice and centrifuged at 16,800× *g* for 20 min following five strokes of a syringe, and the supernatant was used as nuclear fractions.

### 2.7. Western Blot Analysis

Following the treatment under the indicated conditions, cell extracts were prepared with lysis buffer from cell signaling containing protease inhibitor. The lysates were centrifuged at 20,000× *g* for 20 min at 4 °C and used for Western blot analysis. Proteins were separated via SDS-PAGE and then transferred to nitrocellulose membranes. The membranes were blocked for 30 min in 5% (w/v) non-fat skim milk in phosphate-buffered saline (PBS) containing 0.05% Tween-20 (PBST). The blocked membranes were incubated with the indicated antibody for 2 h or overnight at 4 °C. After washing with 1× PBST, the membranes were incubated with either anti-mouse or anti-rabbit horseradish peroxidase (HRP)-conjugated antibody (Thermo Scientific, Rockford, IL, USA) for 1 h, and visualized using the FUSION-SOLO imaging system (Vilber Lourmat, ZAC de Lamirault, France). Antibodies against BAX, Bcl-2, p21, p53, PARP-1, caspase-3, caspase-7, caspase-8, caspase-9, HDAC1, HDAC2, HDAC3, and HDAC8 were purchased from Santa Cruz Biotechnology. Antibodies against acetylated-p53 (K373, K381, and K382) and ERα were obtained from Merck Millipore (Darmstadt, Germany). Anti-β-actin antibodies were bought from Sigma-Aldrich (St. Louis, MO, USA).

### 2.8. RNA Extraction and Quantitative Real-Time PCR

Total RNA was isolated using the RNA Easy-spin kit (Intron Biotechnology Inc., Seongnam-Si, Korea) and reverse transcribed using random primers and the StrataScript reverse transcriptase kit (Stratagene, La Jolla, CA, USA) according to the manufacturer’s instructions. qRT-PCR was carried out using 7500 Real-Time PCR System (Applied Biosystmes, Forster City, CA, USA) with SYBR Green PCR master mix (Thermo Fisher Scientific, Waltham, MA, USA). All reactions were performed in triplicate, and were normalized to glyceraldehyde 3-phosphate dehydrogenase (*GAPDH*). The relative mRNA expression levels were calculated using the comparative quantification method. The primers used in this experiment were as follows: *p21*, 5′–GTGGAGAGCATTCCATCCCT–3′ and 5′–TGGATGCAGCTTCCTCTCTG–3′; p53 upregulated modulator of apoptosis (*PUMA*), 5′–ACTGTGAATCCTGTGCTCTGCC–3′ and 5′–CAAATGAATGCCAGTGGTCACAC–3′; and *ERα*, 5′–TCTACTTTGCCAGCAAACTGGTGC–3′ and 5′–TGTCCAGCCCATGATGGTTCTGAT–3′.

### 2.9. Reporter Assays

Cells were transiently co-transfected with pRL-SV40 (Promega, Madison, WA, USA), and a reporter plasmid with p53 response element (pGL3-p53-RE-Luc) was constructed from the *BBC3* encoding p53 upregulated modulator of apoptosis (PUMA) promoter (−3196 to −2696 bp) bearing the p53 binding site. Total proteins from the cells were extracted, and dual luciferase activity was measured according to the manufacturer’s protocol (Promega, Madison, WA, USA). The *Renilla* (pRL-SV40) luciferase activity was used to normalize all reporter activities. The results were demonstrated as the mean ± SD of three independent experiments.

### 2.10. Cell Apoptosis Assays Using Flow Cytometry

Apoptotic cells were stained using the annexin V-PE/7-AAD apoptosis detection kit (BD Biosciences, CA, USA). Cells were exposed to the indicated conditions, and were collected after 24 h. According to the manufacture’s protocol, the harvested cells were incubated with the anti-annexin V-phycoerythrin (PE) antibody and propidium iodide for 15 min in 1× binding buffer. Subsequently, the apoptotic (annexin V-positive) cells were quantified using a BD fluorescence-activated cell sorting (FACS) Calibur flow cytometer, and the results were analyzed using ModFit LT 2.0 (Verity Software House, Inc., ME, USA).

### 2.11. TUNEL Staining

The possibility of induction of apoptosis by the TNF-α treatment was detected using a 5-bromo-2′-deoxyuridine (BrdU)-Red terminal deoxynucleotidyl transferase dUTP nick end labeling (TUNEL) assay kit (Abcam). A total of 8 × 10^4^ cells were exposed to TNF-α for 24 h, and were stained with ethidium bromide (EtBr) at 37 °C for 1 h. The stained cells were monitored using fluorescence microscopy.

### 2.12. Immunoprecipitation Analysis

Cellular proteins were extracted using lysis buffer. The extracts were incubated with 1 µg of the indicated antibodies and 20 µL of Protein A/G agarose beads (Santa Cruz Biotechnology, TX, USA) overnight at 4 °C. Immunocaptured proteins were washed multiple times with lysis buffer, and the required experiments were performed as described above.

### 2.13. Chromatin Immunoprecipitation Assay

A total of 1 *×* 10^8^ cells were seeded in 15 mm dishes, and when the cells reached approximately 70% confluence, we treated them with or without 20 ng/mL TNF-α for 24 h. A chromatin immunoprecipitation (ChIP) assay was carried out via a modified method based on the manufacturer’s indication from a Pierce Agarose ChIP Kit (Thermo Fisher Scientific, Waltham, MA, USA). Briefly, cells were initially cross-linked in the PBS solution containing 1% formaldehyde for 10 min, which was stopped by adding 125 mM glycine. The cells were washed three times with cold PBS, suspended in SolA buffer (10 mM 4-(2-hydroxyethyl)-1-piperazineethanesulfonic acid (HEPES) (pH 7.9), 1.5 mM MgCl_2_, 10 mM KCl, 0.5% NP-40, and 0.5 mM EDTA) containing a protease inhibitor cocktail (Roche, Branchburg, NJ, USA) by pipetting. After a short spin, the pellets were resuspended in SolB buffer (20 mM HEPES (pH 7.9), 25% glycerol, 0.5% NP-40, 420 mM NaCl, 1.5 mM MgCl_2_, and 0.2 mM EDTA) containing a protease inhibitor cocktail (Roche, Branchburg, NJ, USA), collowed by vigorous pipetting to extract nuclear protein. After centrifugation at 20,000× *g* for 30 min, chromatin was broken by micrococcal nuclease digestion from Pierce (Thermo Fisher Scientific, Waltham, MA, USA) into fragments of 0.5-1.0 kb in average length. The left steps of the ChIP assay after this were performed according to the manufacturer’s protocol of a Pierce Agarose ChIP Kit with the indicated antibodies. The primers used for ChIP assays were as follows: p53 response element of the *p21* promoter, sense 5′-GTGGCTCTGATTGGCTTTCTG-3′, antisense 5′-CTGAAAACAGGCACGGGAAG-3′; p53 response element of the *PUMA* promoter, sense 5′-CGTACATCGGTCGGTCTGTGTACG-3′, antisense 5′-CCAGACACCGGGACAGTCG-3′.

### 2.14. Statistical Analysis

Statistical analysis was carried out using the SPSS software (SPSS Inc., Chicago, IL, USA). Student’s *t*-test was sued to observe the statistical significance between the indicated groups. A value of *p* < 0.05 was considered significant.

## 3. Results

### 3.1. TNF-α Selectively Induced Apoptosis in Human MCF-7 Breast Cancer Cells

**Figure 1:**
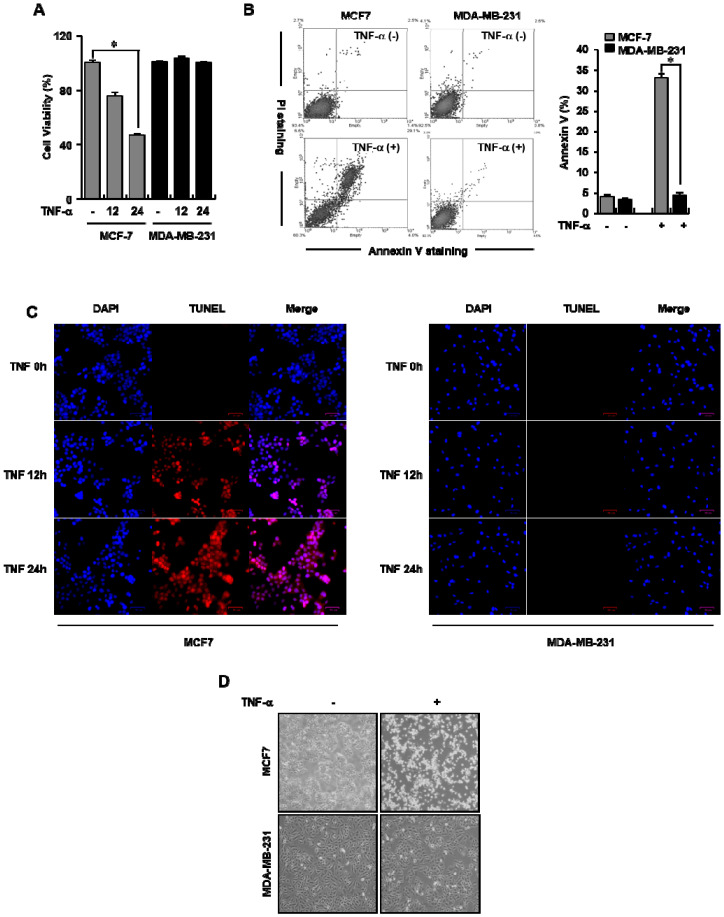
Tumor necrosis factor-α (TNF-α) induced apoptosis in human estrogen receptor-α (ERα)-positive human breast cancer (MCF-7) cells. (**A**) TNF-α selectively reduced the viability of MCF-7 cells. A total of 20 ng/mL of TNF-α was administered for the indicated times to either MCF-7 or MDA-MB-231 cells, and cell viability was assessed using an 3-(4,5-dimethylthiazol-2-yl)-2,5-diphenyltetrazolium bromide (MTT) assay. * *p* < 0.05. (**B**–**D**) TNF-α selectively caused apoptotic cell death in MCF-7 cells. MCF-7 and MDA-MB-231 cells were incubated with TNF-α for 24 h. Subsequently, annexin V/propidium iodide (PI) staining was conducted using a flow cytometer. * *p* < 0.05. Increased apoptosis was detected in fluorescence microscopy images (100×) of 5-bromo-2′-deoxyuridine (BrdU)-Red TUNEL-stained MCF-7 and MDA-MB-231 cells after treatment with TNF-α for 12 and 24 h. Nuclei were stained with 4′,6-diamidino-2-phenylindole (DAPI). The data are represented as the mean ± SD of triplicate measurements. MCF-7 and MDA-MB-231 cells were exposed to TNF-α for 24 h. The cell morphology was visualized using an inverted microscope (100×).

To investigate whether TNF-α triggers apoptosis in breast cancer cell lines, we treated either MCF-7 or MDA-MB-231 cells with TNF-α for the indicated time and adapted various experiments to assess apoptotic cell death. As shown in Figure 1A, TNF-α did not affect the viability of MDA-MB-231 cells at any of the treatment time points, whereas it showed an obvious time-dependent cytotoxic effect in MCF-7 cells compared with in the control group. To test whether the aforementioned cytotoxic effect was related with apoptotic cell death, cleavage of PARP-1, which is considered to be a hallmark of apoptosis, was explored. MCF-7 or MDA-MB-231 cells were exposed to TNF-α for the indicated time, and the indicated protein’s expression was detected using western blot analyses. An increase in the cleavage of PARP-1 after TNF-α treatment was time-dependently detected in MCF-7, but not in MDA-MB-231 cells (Figure S1). To confirm the apoptotic effect of TNF-α, an annexin V/PI double staining assay was performed. In the control group, MCF-7 cells (93.4%) or MDA-MB-231 cells (92.5%) were clustered together in the bottom-left region (negative for both annexin V and PI; AV−/PI−). Cells in the upper-right (AV+/PI–) or bottom-right (AV+/PI+) regions represented those undergoing early and late apoptosis, respectively. The percentage of apoptotic cells among the untreated MCF-7 cells was < 4% compared with MDA-MB-231 cells. However, after TNF-α treatment, only 60.3% of MCF-7 cells remained in the AV−/PI− region, whereas 92.3% of MDA-MB-231 cells remained in the same region. Correspondingly, the percentage of MCF-7 cells in the AV+/PI− and AV+/PI+ regions was significantly increased, indicating an increase in early and late apoptosis, respectively (Figure 1B, left panel). After exposure to TNF-α for 24 h, the annexin V level was significantly increased from 5% to 30% in MCF-7 cells and from 4.8% to 5.2% in MDA-MB-231 cells compared with that observed in control cells (Figure 1B, right panel). Furthermore, the TNF-α-induced apoptotic death of breast cancer cells was also visualized using BrdU-Red TUNEL staining. As shown in Figure 1C, the number of TUNEL-stained MCF-7 cells increased during treatment with TNF-α in a time-dependent manner, whereas the number of TUNEL-stained MDA-MB-231 did not. To validate the TNF-α-elicited apoptosis in breast cancer cells, their morphology was captured using phase-contrast microscopy following TNF-α treatment for 24 h. Unlike the TNF-α untreated MCF-7 cells, treated cells showed symbolic morphological characteristics of apoptosis, such as cell shrinkage and reduced cell density. In contrast, these morphological characteristics were not observed in TNF-α-treated MDA-MB-231 cells (Figure 1D). Taken together, these results indicate that TNF-α significantly induced apoptotic cell death in ERα-positive MCF-7 human breast cancer cells, but not in MDA-MB-231 cells.

### 3.2. TNF-α-Induced Apoptosis in MCF-7 Cells was Dependent on Caspase-7

**Figure 2:**
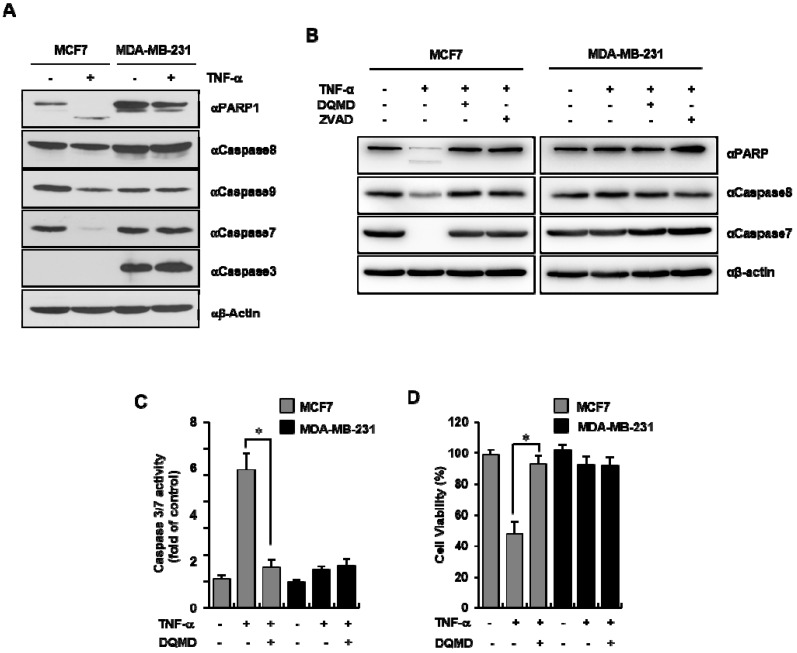
TNF-α-induced apoptotic cell death in MCF-7 was dependent on caspase-7. (**A**) TNF-α induced the activation of effector caspases and the intrinsic apoptotic pathway. MCF-7 or MDA-MB-231 cells were treated with TNF-α (20 ng/mL) for 24 h. The total lysates were analyzed by Western blot analysis using the indicated antibodies. Caspase-8, caspase-9, caspase-7, caspase-3, and poly(adenosine diphosphate (ADP)-ribose) polymerase (PARP)-1 were dramatically converted to the active forms after TNF-α administration to MCF-7 cells; in contrast, there were no significant changes in MDA-MB-231 cells. (**B**) TNF-α-induced apoptosis was suppressed by the caspase inhibitors ZVAD and DQMD. Following pre-treatment of either ZVAD or DQMD for 30 min, MCF-7 and MDA-MB-231 cells were subsequently exposed to 20 ng/mL TNF-α for 24 h. Total cell extracts were used for Western blot analysis using the indicated antibodies. (**C**) TNF-α significantly induced the activation of caspases in MCF-7 cells. Following pre-treatment of 40 μM DQMD for 30 min, MCF-7 and MDA-MB-231 cells were subsequently treated with 20 ng/mL TNF-α for 24 h. The activity of caspase-3/7 was inhibited by the DQMD treatment. * *p* < 0.05. (**D**) TNF-α-induced cytotoxicity was suppressed by the caspase inhibitor DQMD. MCF-7 and MDA-MB-231 cells were pre-treated for 30 min with or without DQMD (40 μM); subsequently, they were treated with TNF-α (20 ng/mL) for 24 h. Cell viability was measured using an MTT assay. All data are presented as the mean ± SD of triplicate measurements. * *p* < 0.05.

To explore further the apoptosis-inducing mechanisms in MCF-7 cells, we determined the expression of the key proteins implicated in apoptosis, as indicated in Figure 2A. MCF-7 and MDA-MB-231 cells were treated with TNF-α for 48 h, and Western blot analysis was carried out. In MCF-7 cells, TNF-α treatment led to a decrease in the expression of caspase-8, caspase-9, and caspase-7, whereas no changes were observed in MDA-MB-231 cells. Moreover, cleavage of PARP-1 was detected in the MCF-7 cells after TNF-α treatment. Interestingly, we found that caspase-3 was not expressed in the MCF-7 cell line. PARP-1 cleavage, which is a major indicator of apoptosis, is mediated by the effector caspases, caspase-3 and caspase-7 [20,21]. Thus, a specific inhibitor of caspase-3/7 (DQMD) was administered to reconfirm the association between caspase-3/7 in TNF-α-induced apoptotic cell death. TNF-α was administered to both cell lines in the absence or presence of DQMD, and caspase-3/7 activity and cell viability were measured. We found that in MCF-7 cells, the increased caspase-3/7 activity observed after TNF-α treatment was dramatically inhibited in the presence of DQMD and that, expectedly, cell viability was almost identical to that of the control group after treatment with the caspase-3/7 inhibitor. No alterations were shown in MDA-MB-231 cells after this treatment (Figure 2A,B). Next, as a result of measuring the activity of actual caspase-3/7 in both cell lines, expectedly, as in the previous results, in the MCF-7 cells the increased caspase-3/7 activity by TNF-α treatment was inhibited, following the DQMD co-treatment to a level similar to that of the control group. However, in the MDA-MB-231 cell line, no changes were found (Figure 2C). Finally, cell viability was measured to observe whether caspase-7 activity was related to MCF-7 cell death. As shown in Figure 2D, following the TNF-α treatment, cell viability was significantly decreased and it was blocked by DQMD in MCF-7 cells. However, no changes were observed in MDA-MB-231 cells. Taken together, the TNF-α-elicited selective cell death in MCF-7 was caspase-7-dependent.

### 3.3. HDAC3 Was Implicated in TNF-α-Induced Apoptotic Cell Death in MCF-7 Cells

**Figure 3:**
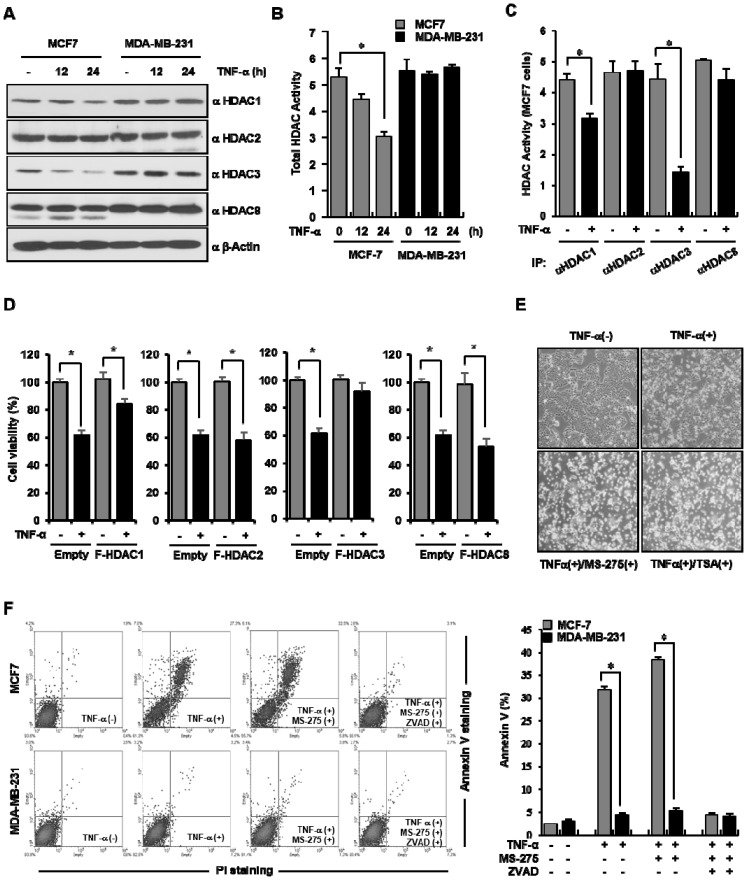
Histone deacetylase 3 (HDAC3) was implicated in TNF-α-induced apoptotic cell death in MCF-7 cells. (**A**) HDAC1 and HDAC3 were downregulated after TNF-α treatment. MCF-7 and MDA-MB-231 cells were treated with TNF-α for the indicated times. The total lysates were analyzed by Western blot analysis using the indicated antibodies. (**B**) TNF-α reduced total HDAC activity. HDAC activity was measured in breast cancer cells after treatment with 20 ng/mL TNF-α for the indicated times. Untreated cells were used as controls. The HDAC assay was carried out using a commercial HDAC assay kit in accordance with the manufacturer’s protocol. The activity of each HDAC was relatively calculated using a TNF-α-untreated group. Values are the mean ± SD of three independent experiments. * *p* < 0.05 (Student’s *t*-test). (**C**) TNF-α selectively inhibited HDAC3 activity. After the treatment of MCF-7 cells with 20 ng/mL of TNF-α, cell lysates were immunoprecipitated with the indicated antibodies. The activities of HDAC-bound beads were measured using a colorimetric HDAC activity assay kit in accordance with the manufacturer’s protocol. Values are the mean ± SD of three independent experiments. * *p* < 0.05 (Student’s *t*-test). (**D**) HDAC3 overexpression inhibited TNF-α-triggered cell death in MCF-7 cells. MCF-7 cells were transfected with plasmids expressing full-length HDAC1, 2, 3, or 8, and TNF-α was administered for 24 h, as indicated. The cell viability was measured using an MTT assay. Values are the mean ± SD of three independent experiments. * *p* < 0.05 (Student’s *t*-test). (**E**–**F**) The inhibition of HDAC activity enhanced TNF-α-induced apoptosis. After pre-treatment with either MS-275, an HDAC1&3 inhibitor or trichostatin A (TSA), a pan-HDAC inhibitor for 30 min, TNF-α (20 ng/mL) was additionally applied to MCF-7 cells for 24 h; cell morphology was then visualized using an inverted microscope (magnification, 100×) (**E**). Following pre-treatment of MS-275 for 30 min, MCF-7 and MDA-MB-231 cells were subsequently treated with TNF-α (20 ng/mL) for 24 h, and then annexin V analysis was performed using a flow cytometer ((**F**), left panel). The percentage of apoptotic cells in the late stage is presented as a bar graph ((**F**), right panel).

Although the roles of HDACs in the control of apoptosis in breast cancer cells have started to be elucidated, the underlying mechanism remains unknown. Thus, to estimate whether HDACs are also associated with apoptosis after TNF-α treatment in breast cancer cells, their expression levels and activities were assessed. First, TNF-α was administered to either MCF-7 or MDA-MB-231 cells for the indicated times, and Western blot analysis was carried out to measure the changes in the expression of class I HDACs. As shown in Figure 3A, in MCF-7 cells, TNF-α treatment destabilized the expression of both HDAC1 and HDAC3 and did not affect the expression of HDAC2 and HDAC8. Furthermore, to understand the biological meaning of the destabilization of HDAC1 and HDAC3 in this setting, we measured total HDAC activity after TNF-α treatment. HDAC activity was decreased after TNF-α treatment in MCF-7 cells alone (Figure 3B). To investigate the impact of TNF-α on each class I HDAC, their activities were measured after immunoprecipitation using the indicated antibodies. Similar to the results presented in Figure 3A, TNF-α selectively decreased the activities of HDAC1 and HDAC3 in MCF-7 cells; in particular, it reduced HDAC3 activity most effectively compared with the activity of the remaining class I HDACs (Figure 3C). Subsequently, to test whether class I HDACs are associated with TNF-α-induced apoptotic cell death in breast cancer cell lines, we examined cell viability after overexpression of each of the class I HDACs. As shown in Figure 3D, the TNF-α induced cell death reported above was blocked by the ectopic overexpression of either HDAC1 or HDAC3 in MCF-7 cells, whereas that of the other class I HDACs (HDAC2 and HDAC8) failed to inhibit apoptotic cell death after TNF-α treatment. Next, to re-confirm the results shown with the involvement of HDAC1 and HDAC3 in breast cancer cell death, we observed the morphological changes using a phase contrast microscope. As shown in Figure 3E, the combination of TNF-α with either MS-275 or TSA significantly induced the characteristic features for apoptotic cell death compared to treatment with TNF-α alone. Finally, in order to solidly confirm not only TNF-α-induced selective cell death in MCF-7 cells, but also the implication of HDAC3 and caspase-7 in this phenomenon, MS-275, a HDAC1 and HDAC3 specific inhibitor, and ZVAD, a pan-caspase inhibitor, were treated alone or in combination with TNF-α in either MCF-7 or MBA-MD-231 cells, followed by FACS analysis after annexin V/PI double-staining assay. As shown in Figure 3F, TNF-α increased apoptotic cell in MCF-7 cells to 31%, but it was hardly observed in MDA-MB-231 cells. The combination of TNF-α with MS-275 enhanced the population of apoptotic MCF-7 cells to 38%, and it was dramatically inhibited following the additional treatment of ZVAD. Taken together, these results suggest that HDAC3 is involved in TNF-α-elicited apoptosis in MCF-7 cells.

### 3.4. HDAC-Mediated ERα Destabilization Caused p53 Activation in TNF-α-Treated MCF-7 Cells

**Figure 4:**
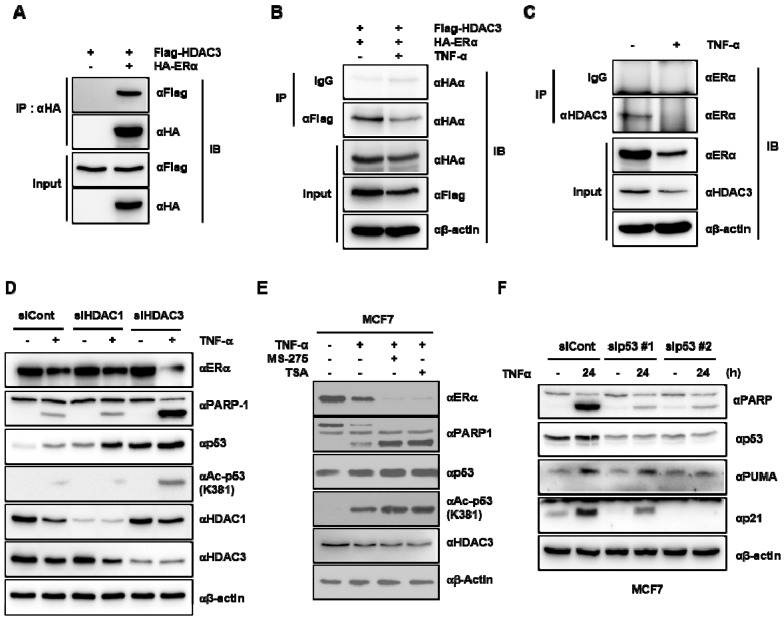
TNF-α-induced decrease in HDAC3–ERα complex formation led to ERα destabilization, consequently enhancing p53 stability and the transcriptional activity of its target gene. (**A**) HDAC3 bound to ERα. Flag-tagged HDAC3 was transfected into MCF-7 cells with either Human influenza hemagglutinin (HA)-empty or HA-tagged ERα vector. Forty-eight hours later, total cell lysates were immunoprecipitated with either an anti-Flag or anti-HA antibody, and the binding between the two proteins was detected by Western blotting using the indicated antibodies. (**B**,**C**) TNF-α induced the reduction of the HDAC3–ERα complex formation. Flag-tagged HDAC3 and HA-tagged ERα were co-transfected into MCF-7 cells, and 20 ng/mL of TNF-α was administered to these cells for 24 h. Total cell lysates were immunoprecipitated with an anti-Flag antibody, and the binding between the two proteins was detected by Western blotting using an anti-HA antibody (**B**). A total of 20 ng/mL of TNF-α was treated in MCF-7 cells for 24 h. Cells were harvested, lysates were immunoprecipitated with the indicated antibodies, and Western blotting was sequentially conducted with an ERα antibody (**C**). (**D**) HDAC3 silencing decreased the expression of ERα and increased p53 stability through its acetylation in TNF-α-treated MCF-7 cells. Either HDAC3 or HDAC1 small interfering RNA (siRNA) was transiently transfected into MCF-7 cells, which were then exposed to TNF-α for 24 h. The total lysates were analyzed by Western blot analysis using the indicated antibodies. (**E**) The inhibition of HDAC activity accelerated ERα destabilization and p53 accumulation. TNF-α was administered together with either MS-275 or TSA to MCF-7 cells. Cells were harvested and lysates were used for Western blot analysis with the indicated antibodies. (**F**) TNF-α-induced selective apoptosis in MCF-7 was p53-dependent. Two sets of 100 pmol p53 siRNA (sip53 #1 or sip53 #2) were transiently transfected in MCF-7 cells, and 20 ng of TNF-α was treated for 24 h. The total cell lysates were analyzed by Western blot analysis using the indicated antibodies.

A previous study demonstrated that the ERα binds directly to the p53 tumor suppressor protein and represses its function. The results reported above suggest that HDAC3 acts as a corepressor complex with ERα for the TNF-α-induced activation of p53 signaling. Thus, we hypothesized that the TNF-α-induced activation of p53 signaling can be attributed to the decrease of the complex formation of HDAC3–ERα, resulting in the activation of the p53 signaling pathway. To test this hypothesis, we first assessed the binding between HDAC3 and ERα. Human influenza hemagglutinin (HA)-tagged ERα was transiently transfected into MCF-7 cells in the presence or absence of Flag-tagged HDAC3, and an immunoprecipitation assay was carried out. As shown in Figure 4A, ectopically overexpressed HDAC3 and ERα bound to each other stably in MCF-7 cells. In confirmation of the results, we observed the reduction of the complex formation after TNF-α treatment through the exogenous or endogenous IP (Figure 4B,C). On the basis of these findings, and to assess whether HDAC3 is responsible for the TNF-α-induced activation of p53, Western blotting assays were carried out after knockdown of HDAC3 using siRNA. As expected, p53 was expressed most stably when HDAC3 was knocked down, together with TNF-α treatment compared with either TNF-α treatment alone or with HDAC1 siRNA treatment alone. The same patterns of HDAC3 action on the expression of p53K381 acetylation and PARP-1 cleavage were observed in the presence of TNF-α treatment (Figure 4D). To confirm this result, the HDAC inhibitors MS-275 (HDAC1/3-specific inhibitor) and TSA (pan-HDAC inhibitor) were applied in the subsequent experiment. As shown in Figure 4E, TNF-α was administered with or without either MS-275 or TSA and the levels of ERα, PARP-1, p53, and p53K381 were observed. Similar to the results depicted in Figure 4E, the levels of PARP-1 cleavage, p53, and p53K381 were higher in the groups that were treated with TNF-α together with either MS-275 or TSA compared with the group treated with TNF-α alone, whereas co-treatment with TNF-α and either MS-275 or TSA dramatically decreased ERα expression compared with TNF-α treatment alone. Lastly, to investigate whether the TNF-α-induced selective apoptosis in MCF-7 cells was p53-dependent, we applied the p53 siRNA system. As shown in Figure 4F, following the TNF-α treatment, PARP-1 cleavage, PUMA, and p21 expressions were significantly decreased in the sip53-treated cells. These data collectively suggest that TNF-α leads to the reduction of the HDAC3–ERα complex formation, ultimately resulting in the activation of the p53 signaling pathway.

### 3.5. TNF-α Triggered the Occupancy Reduction of the HDAC3–ERα and Subsequently Enhanced the Recruitment of the Acetylated p53–p300 Complex to the Promoter Regions in the p53 Target Genes

**Figure 5:**
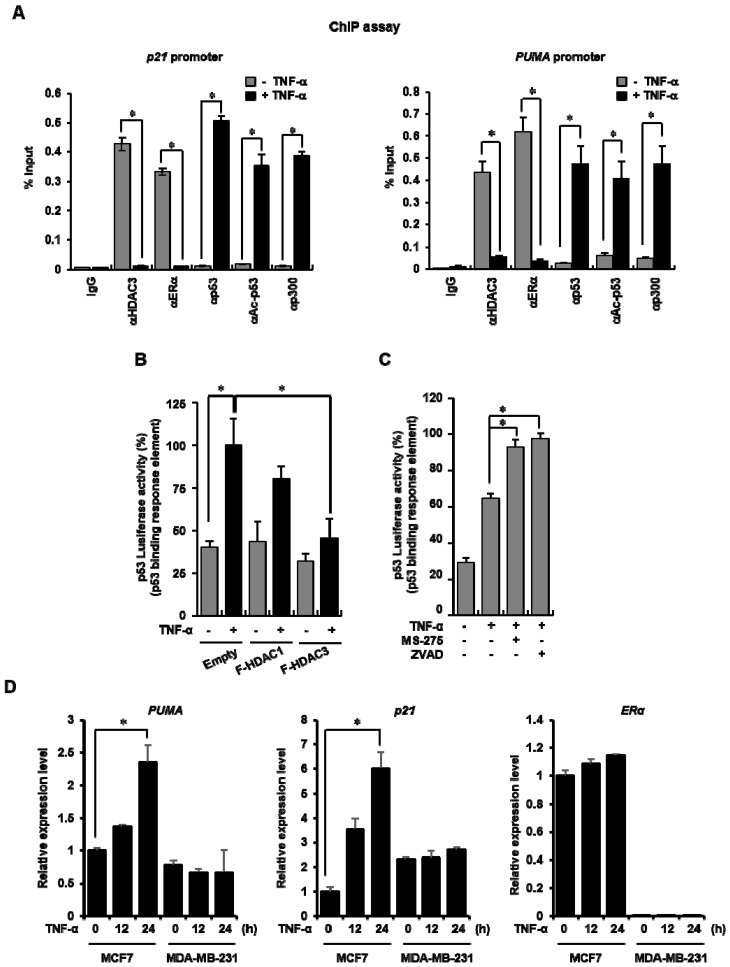
The acetylated p53–p300 complex was replaced with the HDAC3–ERα complex on the *p21* promoter region after TNF-α treatment. (**A**) MCF-7 cells were treated with TNF-α for 24 h, and a chromatin IP (ChIP) assay was conducted with the indicated antibodies. The precipitated DNA were analyzed by qRT-PCR. The values presented are the mean ± SD of three independent experiments. * *p* < 0.05. (**B**) TNF-α-treated MCF-7 cells exhibited increased p53 acetylation. TNF-α was administered to MCF-7 cells for the indicated times. The total lysates were analyzed by Western blot analysis using the indicated antibodies. (**B**) HDAC3 overexpression obstructed the transcriptional activity of the p53 target gene. Reporter assay of p53 binding activity in MCF-7 cells after co-transfection of p53 response element (pGL3-p53-RE-Luc) with either HDAC1 or HDAC3 and treatment with TNF-α for 24 h. The activities of promoters bearing p53-RE were measured in accordance with the manufacturer’s instructions. Values are the mean ± SD of three independent experiments. * *p* < 0.05 (Student’s *t*-test). (**C**) The inhibition of HDAC activity enhanced the transcriptional activity of the p53 target gene. Cells were transfected with pGL3-p53-RE-Luc, pre-treated with either MS-275 or TSA for 30 min, and additionally exposed to TNF-α (20 ng/mL) for 24 h. The activities of the promoters bearing p53-RE were measured in accordance with the manufacturer’s instructions. Values are the mean ± SD of three independent experiments. * *p* < 0.05 (Student’s *t*-test). (**D**) The mRNA expression of apoptosis-related genes, such as p53 upregulated modulator of apoptosis (*PUMA*) and *p21*, was increased in TNF-α-treated MCF-7 cells. Either MCF-7 or MDA-MB-231 cells were exposed to 20 ng/mL of TNF-α for the indicated times. mRNA was extracted from the cells, and the mRNA expression levels of the indicated genes were measured by qRT–PCR. The values presented are the mean ± SD of three independent experiments. * *p* < 0.05.

On the basis of the previous results, we hypothesized that the TNF-α-induced activation of the p53 signaling pathway leads to the decrease in the binding of the HDAC3–ERα complex at the p53 response element of its target genes and results in the activation of p53-mediated transcription, corresponding to the recruitment of the p53–p300 complex to the region. To test this hypothesis, we first examined whether the TNF-α-induced decrease of the HDAC3–ERα complex formation enhanced the recruitment of p53 to the p53-binding sites of its target genes, the *p21* and *PUMA* promoter region. TNF-α treatment efficiently induced the binding of acetylated p53 and p300 to the p53-binding region of both promoter regions, whereas HDAC3 and ERα occupancy on the region of the promoters was significantly diminished after TNF-α treatment (Figure 5A). Next, we assessed the effect of HDAC3 on the transcriptional activity of p53 on the basis of a luciferase activity assay. The plasmid bearing p53 response elements was transfected together with either HDAC1 or HDAC3, followed by treatment with TNF-α, as indicated in Figure 5B. TNF-α treatment led to the dramatic activation the p53 response element. However, HDAC3 overexpression impeded the TNF-α-induced transcriptional activity of the gene. Finally, to confirm the effect of HDAC3 on the transcriptional activity of p53, either MS-275 or TSA was administered together with TNF-α, and a reporter gene assay was carried out. As shown in Figure 5C, the activities were significantly higher in the two groups treated with the HDAC inhibitor together with TNF-α compared with the group treated with TNF-α alone. Finally, the target genes’ transcriptional activity of p53 responsible to apoptosis were observed through the qRT-PCR analysis. As expected, following TNF-α expose, *PUMA* and *p21* mRNA expression increased in MCF-7 cells only. These data collectively demonstrated that the occupancy reduction of HDAC3 and ERα in the p53 response region made the p53–p300 complex recruited after TNF-α treatment more accessible, leading to the induction of p53 target genes in the presence of ERα.

## 4. Discussion

A growing body of evidence has shown that HDAC3 inhibition accelerates p53 acetylation and stability in human cancer cells [26,27]. In breast cancer in particular, ERα is an important factor that plays essential roles in cancer development, progression, and treatment [28,29], and is considered a novel therapeutic target [30]. Furthermore, several studies have shown that the regulation of ERα through HDACs or DNA methyltransferases (DNMTs) [31,32,33] indicates the significance of the epigenetic regulation of ERα in breast cancer. Here, to our knowledge, we observed for the first time that HDAC3 inhibition caused ERα dissociation from the p53-binding element on its target gene in ERα-positive human breast cancer cells, resulting in TNF-α-induced, p53-dependent apoptotic cell death.

The multifunctional TNF-α is implicated in apoptosis. Our data demonstrated the presence of TNF-α-induced selective apoptotic cell death in ERα-positive MCF-7 human breast cancer cells; interestingly, this effect was dependent on caspase-7. A previous study reported that TNF-α promotes FasL expression and causes the acceleration of caspase-3-dependent apoptosis in neuroblastoma cells [34]. Although there is evidence indicating that the apoptosis induced by TNF-α is caspase independent [35], many other studies reported caspase-3-dependent apoptotic cell death in human cancer or normal cells after exposure to TNF-α [36,37,38]. Importantly, we found that caspase-3 was not expressed in MCF-7 cells. In fact, caspase-3 is deficient in MCF-7 cells because of a deletion mutation in exon 3 of the gene [39]. Thus, consistent with two previous studies [39,40], our study showed that caspase-7 was activated after TNF-α administration to MCF-7 cells lacking caspase-3, which raises the possibility that caspase-7 is able to compensate for the lack of caspase-3. Interestingly, the phenomenon of TNF-α-induced activation of caspases was only detected in the ERα-positive MCF-7 human breast cancer cells, and not in the ERα-negative MDA-MB-231 human breast cancer cells. Although this result suggests that ERα is strongly implicated in TNF-α-triggered apoptotic cell death in breast cancer cells, we could not conclusively infer that ERα is a positive regulator of the stimulation of caspase-dependent apoptosis in breast cancer, because our data obviously showed the dramatic decrease in ERα after TNF-α administration to MCF-7 cells, in accordance with a previous study [40]. About 75% of breast cancers are ERα-positive [41]; furthermore, cumulative data from clinical trials [42,43,44] and retrospective data analyses [42] suggest that ERα-positive breast cancer is more tolerant to some chemotherapeutic agents. On the basis of these facts, the downregulation of ERα expression by TNF-α treatment may contribute to overcoming the resistance to chemotherapy in patients with ERα-positive breast cancer; however, the molecular mechanism underlying this phenomenon remains unclear.

Our study demonstrated a possible mechanism mediated by HDAC3 in breast cancer cells. After TNF-α treatment, both HDAC1 and HDAC3 were remarkably downregulated, as was ERα expression, and HDAC activities diminished gradually in a time-dependent manner. Furthermore, by measuring the activity of HDACs through IP using antibodies against each class I HDAC, we determined that the activity of HDAC3 was most obstructed by TNF-α. This result suggests the involvement of HDAC3 in TNF-α-induced apoptotic cell death in ERα-positive breast cancer cells; moreover, other experiments performed in this study using either HDAC3 overexpression or knockdown also firmly supported this hypothesis. As mentioned briefly at the beginning of the Discussion section, there is accumulating evidence of the cross-talk between ERα and HDACs [43,44]. In breast cancer cells, trichostatin A (TSA) and suberoylanilide hydroxamic acid (SAHA), which is a potent and reversible HDAC inhibitor (HDACi), inhibited ERα expression and transcriptional activity [44,45] via the disturbance of HDAC activity. In contrast, the TNF-α-triggered ERα downregulation was attributed to the decline in HDAC3 activity because of its downregulation. Our data demonstrating the ERα distinct fall-off by *HDAC3* knockdown strongly supports the previous results.

Here, the biological significance of the TNF-α-induced HDAC3-mediated downregulation of ERα is an important issue that should be addressed. Lu and colleagues showed that HDAC3 truncation generated by caspase-7 under osmotic stress conditions led to the repression of cell survival and apoptosis [20]. Another study also indicated that HDAC3 degradation after etoposide-induced genotoxic stress promoted p53 function, thus reducing tumor growth [17]. In addition, several studies have shown the importance of HDAC3 in apoptosis progression [21,46,47]. ERα binds directly to the p53 tumor suppressor protein, leading to the downregulation of the transcriptional activation afforded by p53 [25]. In this study, ChIP assays revealed that ERα interacts with the p53-bound promoter of *BRIC5* encoding Survivin in the presence of HDAC4, which are targets for transcriptional repression by p53. Furthermore, ERα knockdown resulted in decreased Survivin expression and enhanced the tendency of MCF-7 cells to undergo apoptosis. Moreover, a previous study showed that ERα inhibited the p53-mediated transcriptional activation of p21, which is involved in apoptosis by recruiting the transcriptional corepressors, nuclear receptor co-repressor (NcoR) and HDAC1 [48]. Our findings combined with these previous results led us to hypothesize that ERα binds to HDAC3 and occupies the p53-binding region of p53 target genes, and that this mechanism is hindered by TNF-α treatment. To substantiate this contention, we performed IP and ChIP assays. Expectedly, ERα formed a specific protein complex with HDAC3, which was co-recruited to the p53-binding element on the *p21* and *PUMA* promoters. Surprisingly, TNF-α administration reduced the complex formation, and a new complex, p300–p53, was positioned on the elements; this implies that apoptotic cell death in ERα-positive breast cancer cells is reversibly modulated by the exchange between the ERα–HDAC3 and p53–p300 complexes. The activation of the *p21* and *PUMA* promoters requires both p53 and p300 [49,50]. P300 also collaborates with Sp1 and Sp3 at the *p21* promoter to facilitate its transcription in a p53-mediated manner [51]. We also found that endogenous p300 expression was dramatically stabilized following the TNF-α treatment (data not shown). This phenomenon not only increased the acetylation of p53, but also stabilized the p53–p300 complex, and could be expected to continuously affect its occupancy in the promoters of the p53 target genes. Considering these reports, the formation of the p53–p300 complex and its occupancy of the promoter of the p53 target gene after TNF-α-induced occupancy reduction of ERα–HDAC3 complex was perhaps predictable.

However, the regulation of p53-dependent apoptotic cell death in MCF-7 cells is unlikely to be driven solely by the recombination of the above-mentioned complexes and their positioning at the *p21* promoter. Consistent with previous studies, our data showed an increase in p53 acetylation and its stabilization via the inhibition of HDAC3 through either siRNA or treatment with an inhibitor. HDAC3 binds to the DNA-binding domain of p53 and inactivates it through deacetylation [17,52,53]. Another HDAC class I protein, HDAC1, binds directly to p53 via the 30 C-terminal regulatory residues of p53 [52], causing p53 inactivation in the same manner as that induced by HDAC3. Surprisingly, this same region of p53 has been identified as the domain that interacts with ERα [24,54,55]. As either HDAC3 or HDAC1 can bind directly to ERα [56], ERα may expedite the association between either HDAC1 or HDAC3 and p53, which may facilitate the deacetylation and inactivation of p53. On the basis of these results, although the TNF-α-induced selective apoptotic cell death in ERα-positive breast cancer cells could be partially analyzed, further in-depth experiments should be carried out to address these plausible and interesting scenarios.

**Figure 6:**
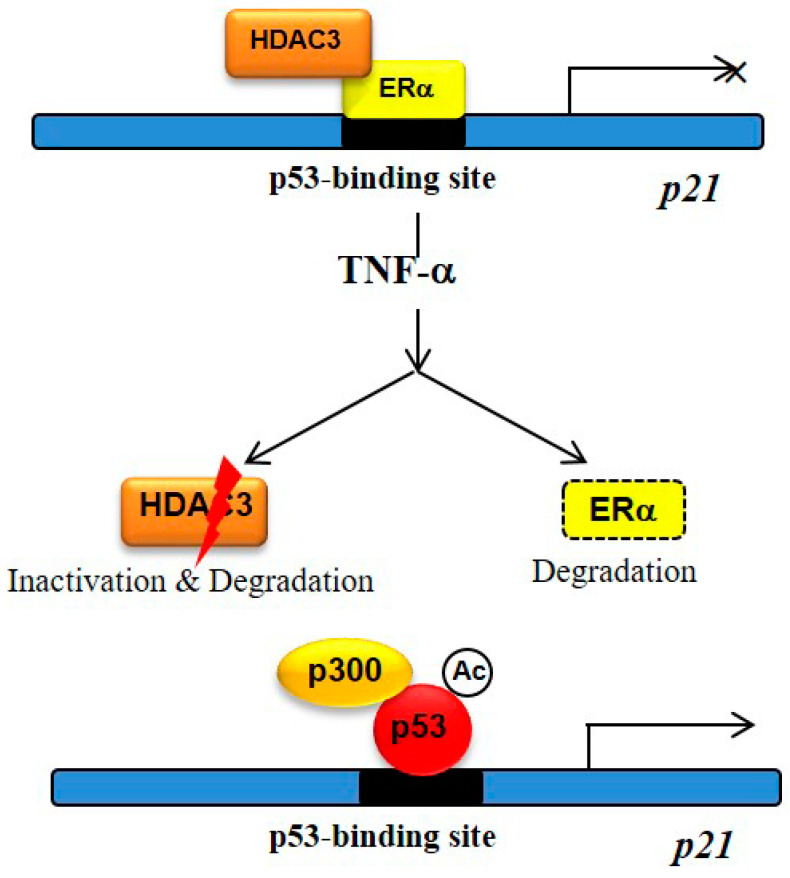
Model of our findings. HDAC3 regulated TNF-α-triggered apoptosis in the ERα-positive MCF-7 human breast cancer cells via the activation of the p53 signaling pathway. The HDAC3–ERα heterocomplex was formed, occupying the p53-binding site of the *p21* promoter, thus blocking its mRNA expression. TNF-α treatment led to the dissociation of the HDAC3–ERα complex. P53 bound to p300 and stabilized it through acetylation modification; subsequently, the acetylated-p53–p300 complex replaced the HDAC3–ERα complex at the p53-binding site of the *p21* promoter, thus enhancing the transcriptional activity of *p21*.

Taken together, our results showed that the ERα–HDAC3-mediated p53-dependent apoptotic cell death in the ER-positive MCF-7 human breast cancer cells was regulated by very complex and diverse mechanisms (Figure 6). However, we demonstrated a plausible novel mechanism through which HDAC3 selectively regulated apoptotic cell death in MCF-7 cells. The findings pertaining to the role of HDAC3 in breast cancers differing in ERα status provide worthwhile information on the clinical relevance of the association among these proteins and the development of new therapeutic and prevention strategies against ERα-positive breast cancer.

## Supplementary Materials

The following are available online at https://www.mdpi.com/2073-4409/9/5/1280/s1, Figure S1: TNF-α-induced PARP1 cleavage was selectively shown in MCF7 cells. MCF-7 or MDA-MB-231 cells were treated with TNF-α (20 ng/mL) for 24 h. The total lysates were analyzed by wwestern blot analysis using the indicated antibodies. The poly(adenosine diphosphate (ADP) -ribose) polymerase (PARP)-1 were dramatically converted to the active forms after TNF-α administration to MCF-7 cells; in contrast, there were no significant changes in MDA-MB-231 cells.
