# Supplementary material for: HDAC3–ERα Selectively Regulates TNF-α-Induced Apoptotic Cell Death in MCF-7 Human Breast Cancer Cells via the p53 Signaling Pathway
Source: Cells. 2020 May 21;9(5):1280. doi: 10.3390/cells9051280 (PMC7290399; doi:10.3390/cells9051280)
Supplement: Supplementary file 1 [file cells-09-01280-s001.zip › cells-773367-supplementary.pptx]

## Slide 1
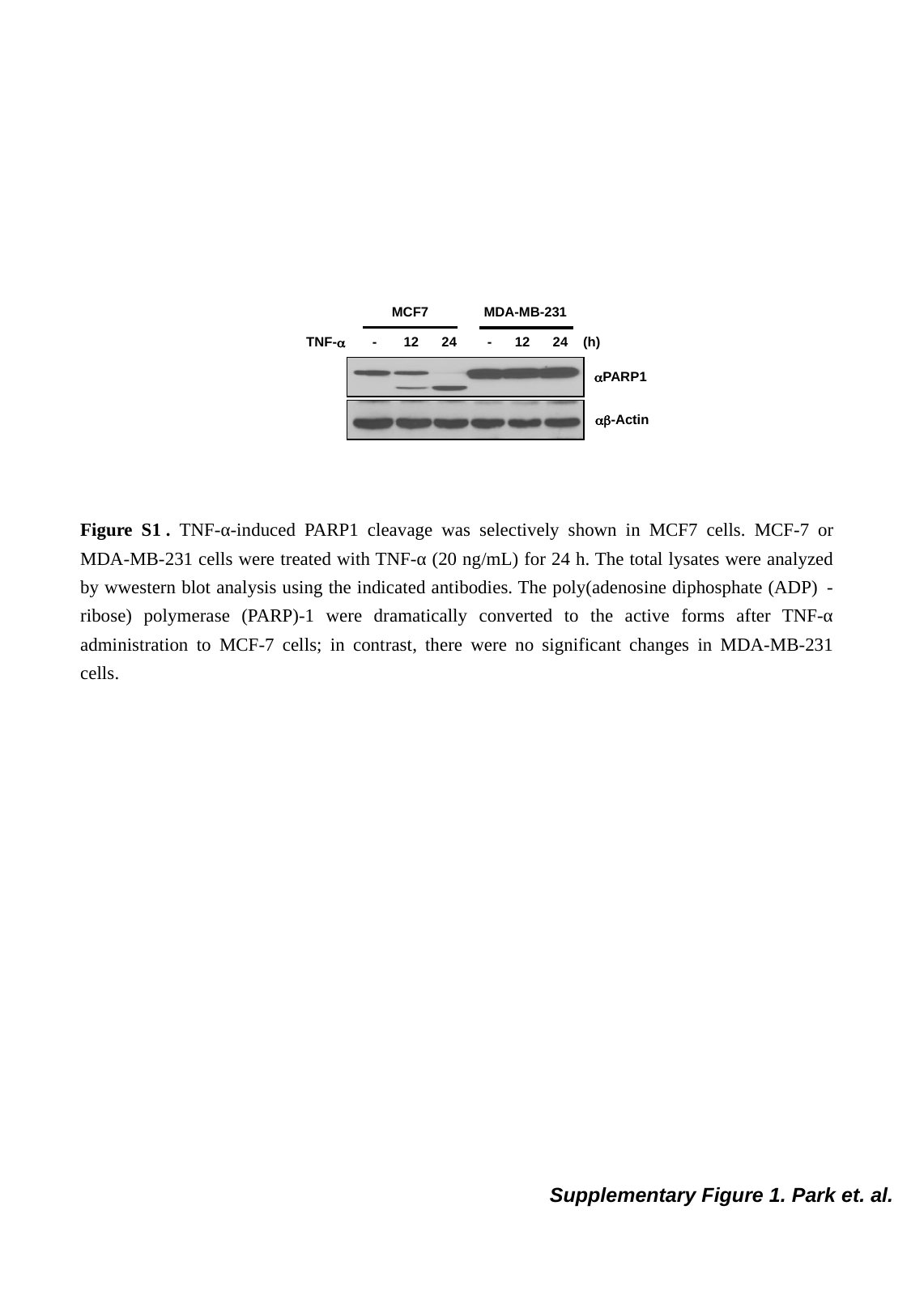

MCF7
MDA-MB-231
TNF-α - 12 24 - 12 24 (h)
αPARP1
αβ-Actin
Figure S1 . TNF-α-induced PARP1 cleavage was selectively shown in MCF7 cells. MCF-7 or MDA-MB-231 cells were treated with TNF-α (20 ng/mL) for 24 h. The total lysates were analyzed by wwestern blot analysis using the indicated antibodies. The poly(adenosine diphosphate (ADP)  -ribose) polymerase (PARP)-1 were dramatically converted to the active forms after TNF-α administration to MCF-7 cells; in contrast, there were no significant changes in MDA-MB-231 cells.
Supplementary Figure 1. Park et. al.
